# Inhibitory effect of *O*-propargyllawsone in A549 lung adenocarcinoma cells

**DOI:** 10.1186/s12906-023-04156-9

**Published:** 2023-09-20

**Authors:** Edmilson Willian Propheta dos Santos, Rauan Cruz de Sousa, Mariana Nobre Farias de Franca, Jileno Ferreira Santos, Flaviano Melo Ottoni, Raquel Geralda Isidório, Waldecy de Lucca Junior, Ricardo José Alves, Ricardo Scher, Cristiane Bani Corrêa

**Affiliations:** 1 Laboratory of Biology and Immunology of Cancer and Leishmania, Department of Morphology, Federal University of Sergipe, São Cristóvão, Sergipe, Brazil; 2 Graduate Program in Health Sciences, Federal University of Sergipe, Aracaju, Sergipe, Brazil; 3 Laboratory of Pharmaceutical Chemistry, Department of Pharmaceutical Products, Faculty of Pharmacy, Federal University of Minas Gerais, Belo Horizonte, Minas Gerais, Brazil; 4 Laboratory of Molecular Neuroscience of Sergipe, Department of Morphology, Federal University of Sergipe, São Cristóvão, Sergipe, Brazil

**Keywords:** Lawsone, *O*-propargyllawsone, Cancer, Cell death, Lung adenocarcinoma

## Abstract

**Background:**

Lung cancer is the deadliest type of cancer in the world and the search for compounds that can treat this disease is highly important. Lawsone (2-hydroxy-1,4-naphtoquinone) is a naphthoquinone found in plants from the *Lawsone* genus that show a high cytotoxic effect in cancer cell lines and its derivatives show an even higher cytotoxic effect.

**Methods:**

Sulforhodamine B was used to evaluate the cytotoxic activity of compounds on tumor cells. Clonogenic assay was used to analyze the reduction of colonies and wound healing assay to the migratory capacity of A549 cells. Apoptosis and necrosis were analyzed by flow cytometer and Giemsa staining. Hemolysis assay to determine toxicity in human erythrocytes.

**Results:**

Lawsone derivatives were evaluated and compound 1 (*O*-propargyllawsone) was the one with the highest cytotoxic effect, with IC_50_ below 2.5 µM in A549 cells. The compound was able to reduce colony formation and inhibit cell migration. Morphological changes and cytometry analysis show that the compound induces apoptosis and necrosis in A549 cells.

**Conclusions:**

These results show that *O*-propargyllawsone show a cytotoxic effect and may induce apoptosis in A549 cells.

**Supplementary Information:**

The online version contains supplementary material available at 10.1186/s12906-023-04156-9.

## Background

Lung cancer is one of the cancers with the highest incidence in the world, leads the first position in mortality and represents a total of 18% of the causes of death from cancer [1]. Tobacco is the main cause of lung cancer and about 80% of cases are associated with its use [2]. The adenocarcinoma type comprises about 60% and it is the most common type of lung cancer in non-smokers [3, 4]. This type is highly aggressive due to a high propensity to metastasize to the brain, liver, bone marrow and adrenal glands [5]. Furthermore, lung cancer develops chemoresistance mechanisms to the drugs used which difficult its treatment [6]. In this context, new treatment strategies are needed and some studies show that natural products have the potential to treat resistant tumors [7].

Medicinal plants are a great source of compounds with biological activity. These compounds show great potential for the treatment of cancer [8–10]. About 65% of approved drugs with antitumor activity are natural products or derivatives of natural products [11]. Several classes of compounds present in plants such as quinones, terpenes and alkaloids are the source of chemotherapeutics that are already used in the treatment of cancer [12, 13].

Lawsone is a quinone, which belongs to the naphthoquinone class, and has been shown to have a great antibacterial, antifungal and antitumor activity [14–16]. It is the main component of the *Lawsonia inermis* Linn species, a plant popularly known as henna [17]. As previously reported, lawsone was capable of inhibiting cell growth of breast cancer (MCF-7), ovarian cancer (SKOV-3) and colon cancer (DLD-1) through enhanced expression of *p53* and reducing NF-kB activity [17–19]. Studies with synthetic derivatives of lawsone show an increase in cytotoxic activity in tumor cell lines [20, 21]. For example, glycosidic derivatives of lawsone are more cytotoxic compared to lawsone against several types of breast cancer (SKBR-3, MDA-MB-231 and MCF-7) [22]. Besides, lawsone derivatives are also highly cytotoxic against doxorubicin-resistant leukemia cells (CEM/ADR5000) [23]. Lawsone stands out for being an important source for the synthesis of new compounds [22].

Recently, we described [24] the synthesis of classical glycosides and glycosyl triazoles derivatives of lawsone and their activity against melanoma (B16-F10), glioma (C6) and lung adenocarcinoma (A549) cancer cell lines. Lawsone, the starting material for the synthesis of the classical glycosides and *O*-propargyllawsone, starting material for the synthesis of the glycosyl triazoles as well as its isomer 3-*C*-propargyllawsone and two non-glycosidic triazole derivatives of lawsone were included in the study for comparison purposes. In the preliminary screening the percentage of cell growth inhibition at a single concentration of 25 μM of all compounds was determined as a guide to select the more interesting compounds for IC_50_ determination and further studies. Although the study was directed to the carbohydrate-based derivatives of lawsone, we noticed that *O*-propargyllawsone displayed one of the highest growth inhibitions. This leaded us to study this compound more deeply and our results are disclosed in this paper which shows the potential of *O*-propargyllawsone in inhibiting cell proliferation and death induction in A549.

## Methods

### Reagents

Dulbeccos's Modified Eagle Medium (DMEM), Trypsin, Triton X-100, Dimethyl sulfoxide (DMSO) and Sulforhodamine B (SRB) from Sigma Aldrich (Saint Louis, MO, USA), Fetal Bovine Serum (FBS) and antibiotic (penicillin 10,000 U/mL; streptomycin 10,000 mg/mL) from Gibco (Life Technologies, India), EDTA (Promega Corporation, Madson U.S.A.), Doxorubicin Hydrochloride (Rubidox, Bergamo), Trichloroacetic acid and Giemsa (NEON), Acetic acid (Synth), Tris (Inlab Trust, Brazil), Propidium iodide, AnnexinV and Accutase (Invitrogen, Life Technologies, India).

### Lawsone derivatives

**Fig. 1:**
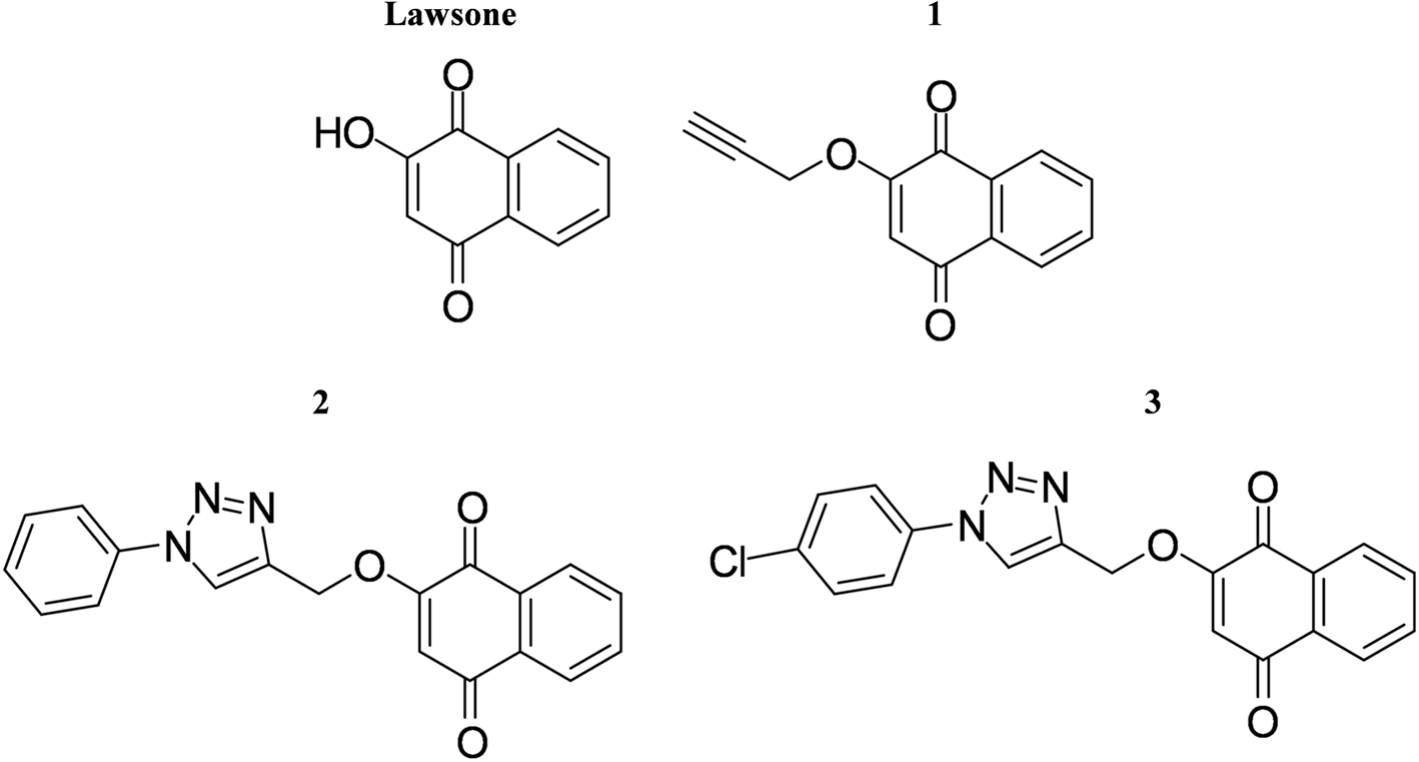
Compounds. Molecular structure of lawsone and compounds 1, 2 and 3

Lawsone and its synthetic derivatives were obtained through a partnership made with PhD Ricardo José Alves from the Pharmaceutical Chemistry Laboratory of the Pharmacy Course at the Federal University of Minas Gerais. Lawsone derivatives compounds 1, 2 and 3 (Fig. 1) were selected for this work due to the similarity of the chemical structure. Thus, stock solutions of 100 mM in Dimethyl sulfoxide (DMSO) of the synthetic derivatives of lawsone were prepared to be used in the in vitro experiments.

### Cell Lines

The cell lines of lung adenocarcinoma (A549), melanoma (B16-F10) and glioma (C6) were purchased from the Federal University of Rio de Janeiro. Cells were maintained in cell culture flasks in Dulbeccos's Modified Eagle Medium (DMEM) supplemented with 10% Fetal Bovine Serum (FBS) and 1% antibiotic (10,000 U/mL penicillin; 10,000 mg/mL streptomycin) and incubated at 37ºC in an atmosphere of 5% CO_2_. After reaching the necessary confluence for carrying out the experiments, the medium was removed, and the cells detached from the bottle using a 0.25% Trypsin–EDTA solution for a period of 1 to 15 min. After this step, the Trypsin–EDTA solution was inactivated with culture medium and the cells were centrifuged, with rotation of 153 G for 10 min at 4ºC. The pellet formed was resuspended in culture medium and the cells were counted in a Neubauer chamber to carry out the experiments.

### Sulforhodamine B assay

For this experiment, A549, B16-F10 and C6 cells were seeded in 96-well plates at a density of 1 × 10^4^ cells/well. After an incubation period of 24 h in a humidified incubator at 37ºC with 5% CO_2_ atmosphere for adhesion, the cells were treated with lawsone and lawsone derivatives (compounds 1, 2 and 3) for 72 h with concentrations from 25 µM to 0.8 µM. Cells were fixed with 100 μL of 30% trichloroacetic acid for 1 h in a refrigerator at 4ºC. Then, the plate was washed 4 times with water and set to dry. After the drying period, 100 μL of 0.057% Sulforhodamine B (SRB) solution (w/v) dissolved in 1% acetic acid was added for 30 min at room temperature. Then the plate was washed 4 times with 1% acetic acid to remove excess dye. After drying, 200 μL of TRIS 10 mM pH 10.5 was added to dilute the SRB for 30 min. The reading was made in a spectrophotometer (Biotek—H1 Synergy) with a wavelength of 510 nm.

Based on the inhibitory degree of the derivatives, the IC_50_ of the compounds were calculated. The IC_50_ is the concentration capable of inhibiting 50% of cell growth [25]. The compound that presented the lowest IC_50_ was compound 1, being selected for evaluation in the following experiments. For this, IC_50_ values ​​were used as a basis to define concentrations of half of the IC_50_, the IC_50_ value and twice the IC_50._

### Giemsa staining

A549, B16-F10 and C6 cells were seeded on coverslips in 24-well plates at a density of 2 × 10^4^ cells/well. After 24 h, cells were treated with the compound 1 derivative using 1.1, 2.2 and 4.4 μM for A549 and C6, and 0.75, 1.5 and 3 µM for B16-F10 for 24 h. After treatment, cells were washed twice with phosphate-buffered saline (PBS) and fixed with methyl alcohol for 3 min. Then cells were stained with Giemsa for 1 min. The slides were evaluated under an optical microscope (Olympus) and the images were captured at 400 × magnification.

### Hemolytic assay

The hemolytic activity of the compound 1 was tested using human erythrocytes from healthy volunteers. Erythrocytes were seeded into 96-well plates (2% per well in 0.2 ml of 1X PBS) and exposed to concentrations of 13.75, 27.5, 55 and 110 µM, the highest concentration tested was 50 times the IC_50_. Triton X 100 (0.1%) and Dimethyl sulfoxide (0.1%) were used as positive and negative hemolysis controls, respectively. Erythrocytes were kept under agitation at a temperature of 37 °C for 1 h. After this time, the plate was centrifuged at 405 × g for 5 min and 0.15 mL of the transferred supernatant was read in a spectrophotometer (Biotek—H1 Synergy) with an absorbance of 540 nm.

### Clonogenic assay

A549 cells were seeded in a 6-well plate at a density of 150 cells/well and incubated in an incubator with 5% CO_2_ atmosphere at 37 °C. After 24 h, cells were treated with the compound 1 at concentrations of 1.1, 2.2 and 4.4 µM for 72 h. Dimethyl sulfoxide 0.02% and Doxorubicin 0.2 μM were used as negative control and positive control respectively. The treatment was removed and replaced with a supplemented DMEM medium. The cells were incubated in an atmosphere of 5% CO_2_ at 37ºC. After 6 days, cells were fixed with methanol + acetic acid (3:1) for 5 min and stained with 0.5% crystal violet in water for 30 min. The plates were photographed, and the images were analyzed using ImageJ 1.46 software.

### Wound-healing assay

A549 cells were seeded in a 12-well plate at a density of 4 × 10^5^ cells/well and incubated in an incubator with 5% CO_2_ atmosphere at 37 °C for 24 h. After the incubation period, a straight-line wound was made with the aid of a p200 tip, the supernatant was discarded, and the wells were washed twice with 1 × PBS to remove loose cells. Cells were treated with compound 1 at concentrations of 1.1, 2.2 and 4.4 µM. 0.02% Dimethyl sulfoxide and 0.2 μM Doxorubicin were used as a negative control and positive control, respectively. Images of wound closure were acquired at 0, 24 and 48 h of treatment with an Olympus microscope. The images were analyzed using the ImageJ software version 1.46.

### Annexin V-FITC/PI assay

A549 cells were seeded in 12-well plates in DMEM medium supplemented with FBS and 1% antibiotic (10,000 U/ml penicillin; 10,000 mg/ml streptomycin) with a density equal to 2 × 10^5^ cells/well and incubated for 24 h. Then, the cells were treated with the compound 1 at concentrations of 1.1, 2.2 and 4.4 µM, for 24 h. Dimethyl sulfoxide 0.02% was used as negative control and 0.2 μM Doxorubicin were used as positive control. After treatment, cells were detached with Accutase solution, transferred to tubes, and washed with PBS. After washing the cells were stained with Annexin V-FITC/PI according to the manufacturer. Samples were analyzed by flow cytometry (Attune NxT Acoustic Focusing Cytometer, Thermo Fisher Scientific). Cells without labeling were considered viable, cells positive only for Annexin V/FITC were considered cells in apoptosis, cells with double labeling for Annexin V/FITC and propidium iodide were considered cells in late apoptosis and cells positive only for iodide propidium were considered cells in necrosis [26].

### Statistical analysis

All results were evaluated by performing three independent experiments, except the Giemsa assay which only two experiments were performed. For all experiments, *p* values ​​ < 0.05 were considered statistically significant. Analyzes and graphs as well as IC_50_ were obtained using the GraphPad Prism 8 program. Shapiro–Wilk normality test was applied to assess the normal distribution of the data. Statistical analysis was made by running ANOVA.

## Results

### Lawsone derivatives show a high percentage of growth inhibition

**Fig. 2:**
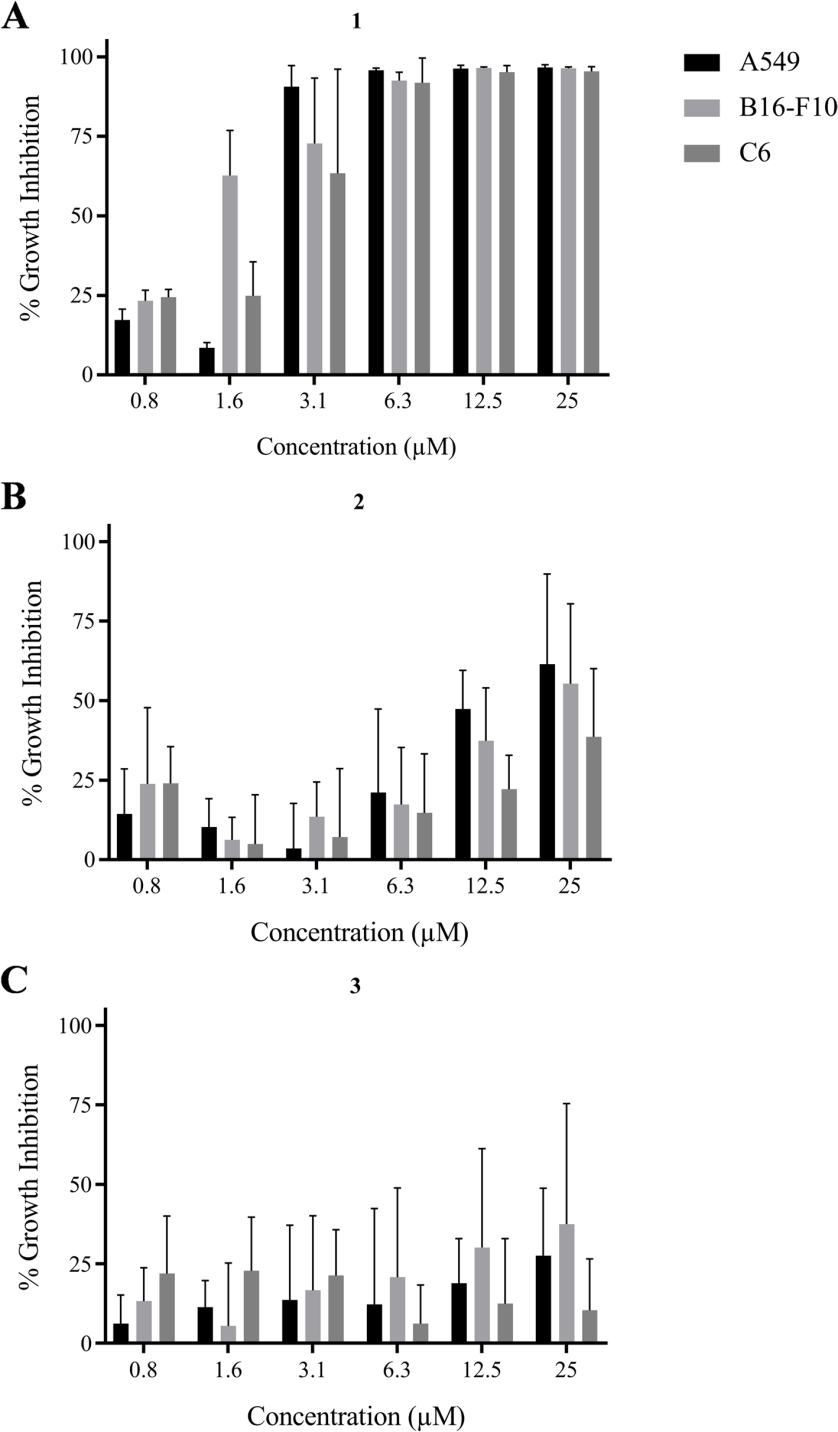
Percentage of growth inhibition of lawsone synthetic derivatives. Results obtained after 72 h treatment with compounds 1 (**A**), 2 (**B**) and 3 (**C**) in A549, B16-F10 and C6 cell lines. The graph represents the mean and standard deviation of three independent experiments

Lawsone derivatives compounds 1, 2 and 3 were used to assess the potential to inhibit the growth of lung carcinoma (A549), melanoma (B16-F10) and glioma (C6) cell lines (Fig. 2). Data of percentage of growth inhibition from Lawsone and Doxorubicin see Additional file 1. In this work, we used the classification described by Mahmoud et al., 2011 which categorizes the inhibitory activity as: high inhibition when above 75%, medium between 75 and 50% and low when below 50% [27]. Compound 1 showed a high inhibition in A549, B16-F10 and C6 cells at concentrations from 25 to 6.3 µM (Fig. 2A). Compound 2 at concentrations 12.5 to 0.8 µM showed a low inhibition for the three cell lines studied and a medium inhibition for A549 and B16-F10 at a concentration of 25 µM (Fig. 2B). Compound 3 showed a low inhibitory effect in all the cell lines at all concentrations tested (Fig. 2C).

The results of the SRB were used to calculate the maximum inhibitory concentration of 50% of cell growth (IC_50_) which are shown in Table 1. Compound 1 was the most cytotoxic compared to lawsone and compounds 2 and 3, with IC_50_ values ​​below 2.2 µM in the three cell lines. The compound 2 showed IC_50_ above 14 µM in the cell lines tested and the IC_50_ values for compound 3 could not be determined since none of the concentrations tested was able to inhibit cell growth by 50%. Lawsone showed IC_50_ values ​​of 22.6 µM for C6 and 53 for A549 cell lines. Doxorubicin, a compound used as a positive control, showed an IC_50_ below 0.6 µM in the A549 and B16-F10 cell lines (Table 1). Considering that compound 1 had the lowest IC_50_ among the compounds tested, it was selected for the evaluation of its activity in the other experiments.

**Table 1:**
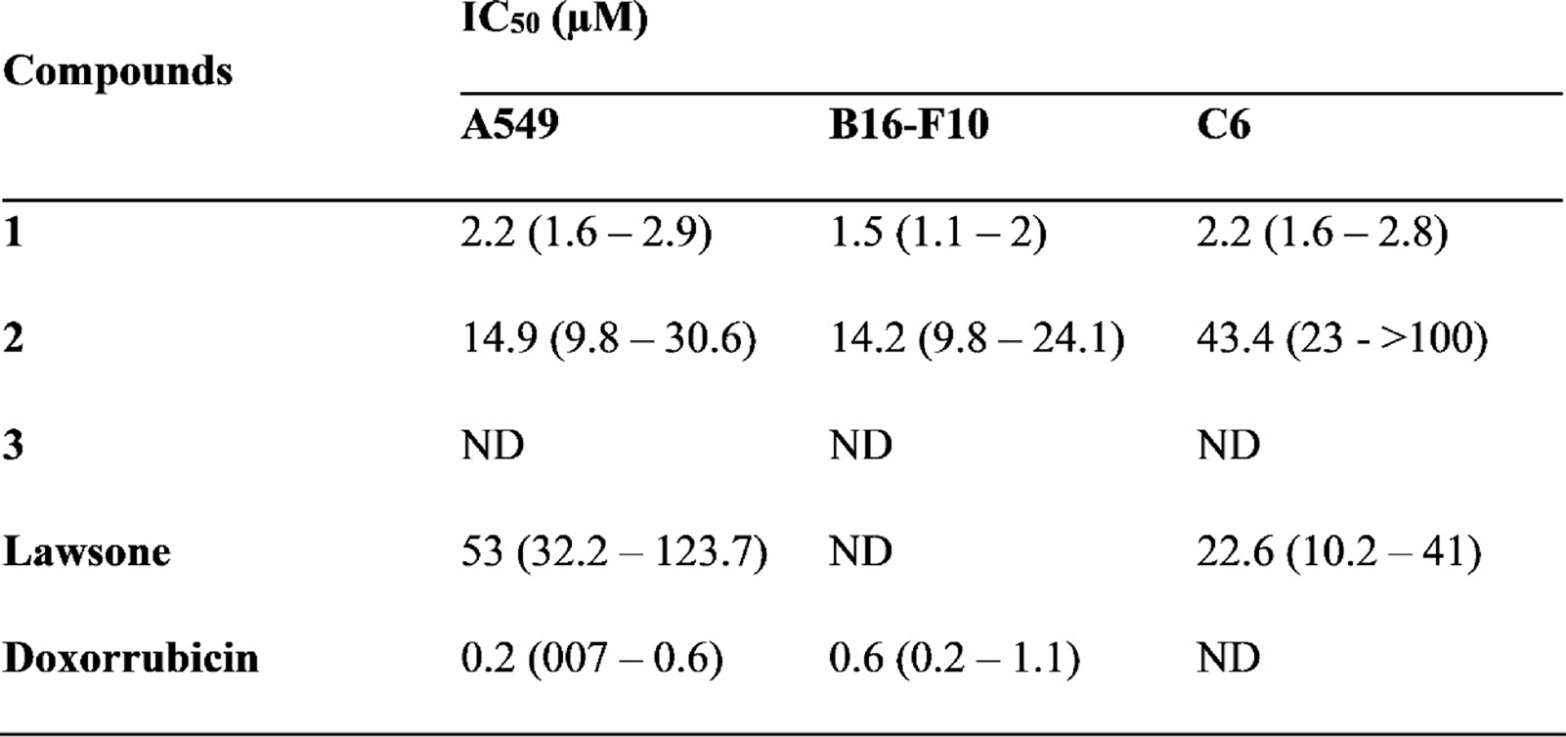
IC50 of compounds 1, 2 and 3. Data in the table show the maximum 50% inhibitory concentration of cell growth (IC50) and the 95% confidence interval

*ND*  Not determined

### Compound 1 induces morphological changes similar to apoptosis

**Fig. 3:**
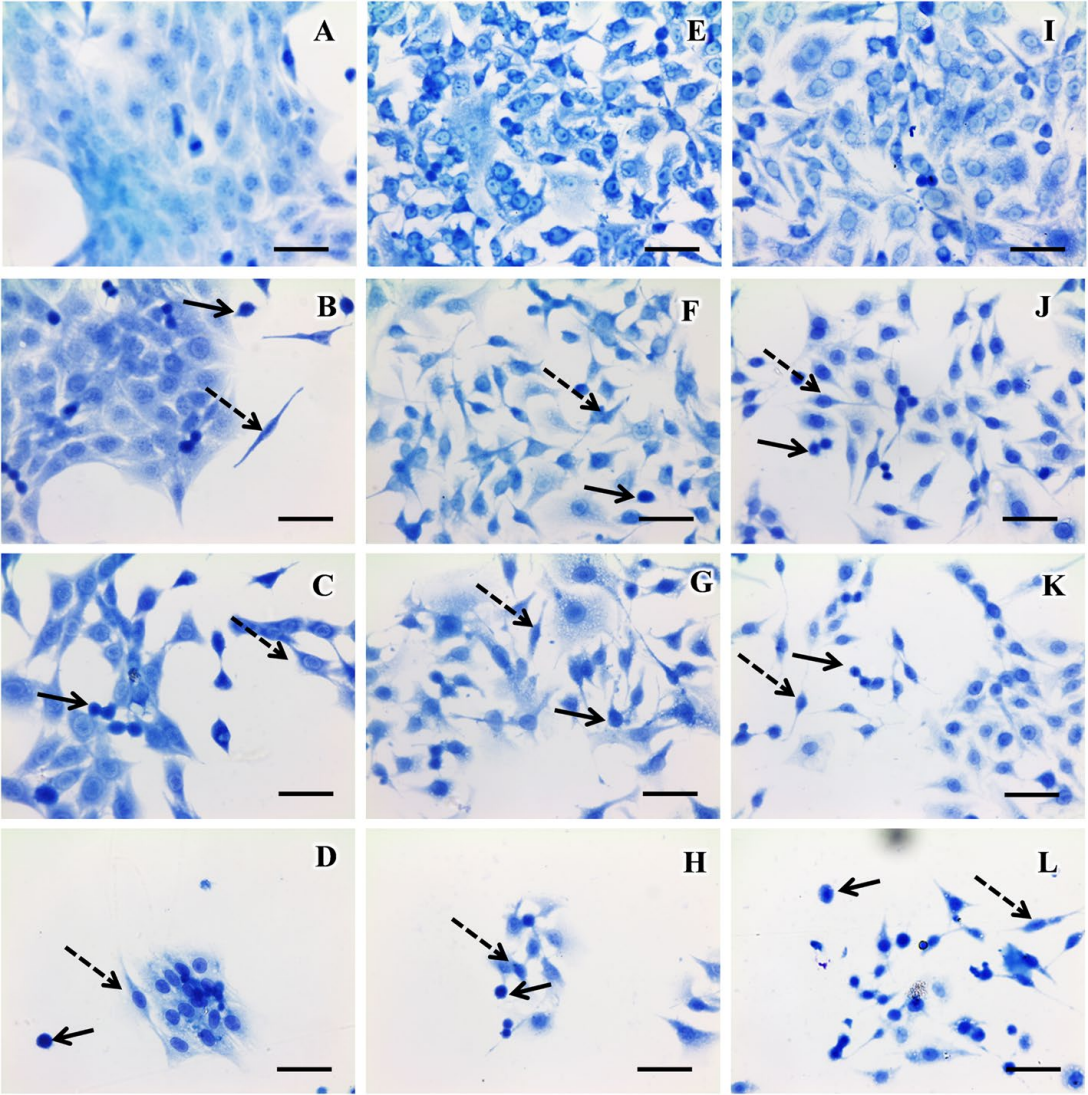
Morphological aspects of the cell lines after 24-h treatment with compound 1. Cells were treated with different concentrations of Compound 1. **A** – A549 cells without treatment. **B**-**D** – A549 cells treated with compound 1 in the concentrations of 1.1, 2.2 and 4.4 µM respectively. **E** – B16-F10 cells without treatment. **F**–**H** – B16-F10 cells treated with compound 1 in the concentrations of 0.75, 1.5 and 3 µM respectively. **I** – C6 cells without treatment. **J**-**L** – C6 cells treated with compound 1 in the concentrations of 1.1, 2.2 and 4.4 µM respectively. Solid arrow indicates the position of apoptotic cells and dotted arrows indicate the position of cells with reduced cytoplasm. Bar—50 µm. Representation of two independent experiments

For the analysis of morphological changes, compound 1 was used in concentrations referring to 0.5xIC_50_, 1xIC_50_ and 2xIC_50_ in A549, B16-F10 and C6 cell lines. Therefore, the concentration used for treatment were 1.1, 2.2 and 4.4 µM to A549 and C6 cell lines and 0.75, 1.5 and 3 µM to the B16-F10 cell line (Fig. 3). After 24 h treatment with compound 1 at a concentration of 1.1 and 0.75 µM, it was possible to observe morphological changes such as reduced cytoplasmic volume and the appearance of apoptotic cells in the C6 and B16-F10 lines, respectively (Fig. 3J and F). At the concentrations of 2.2 and 4.4 μM for A549 and C6 and 1.5 and 3 µM for B16-F10 it was possible to observe a reduction in cytoplasm and apoptotic cells for the A549, B16-F10 and C6 cells (Fig. 3C, D, H, K and L). The results observed in the cytotoxicity assay and morphological analysis show that compound 1 has a high cytotoxic effect in the A549 cell line. Therefore, based on the cytotoxic effect and the highest incidence of lung cancer in the world, compound 1 was selected for the next assays in the A549 cells.

### Compound 1 shows low toxicity in human erythrocytes

**Fig. 4:**
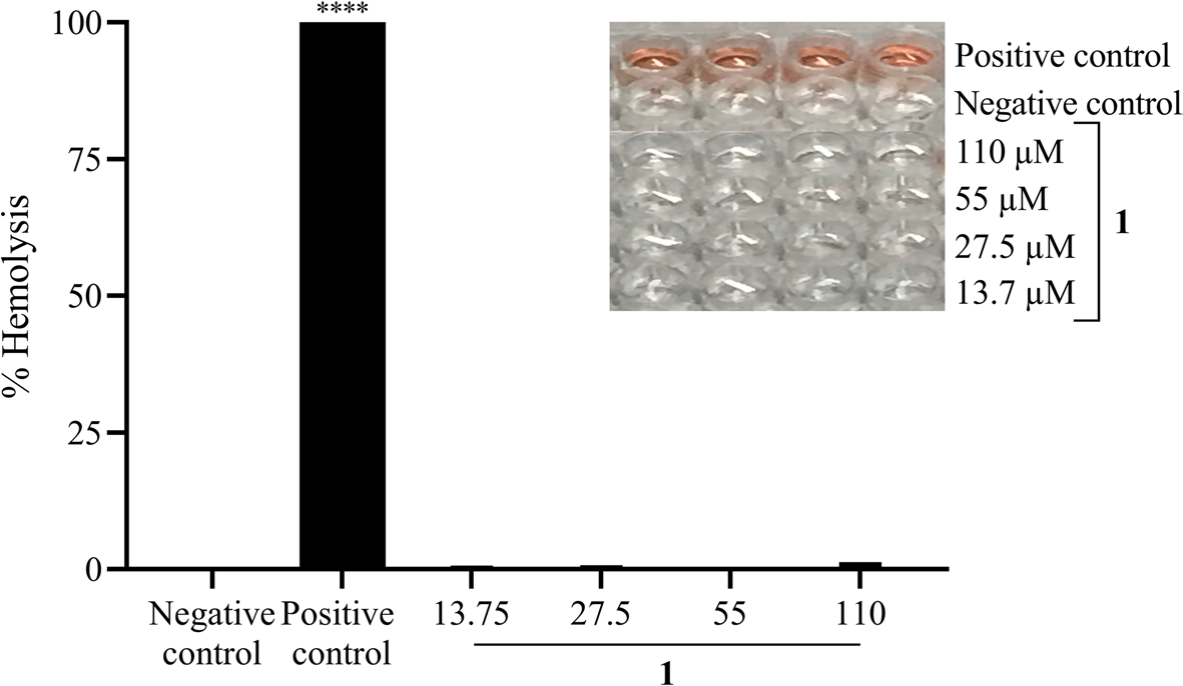
Percentage of hemolysis after treatment with compound 1. The graphics show the results of the Hemolysis assay after treatment with compound 1 with the concentrations of 13.75, 27.5, 55 and 110 µM for 1 h. Triton-X100 positive control and DMSO 0.1% negative control. Data were submitted to One-way ANOVA followed by Dunnett's post-test was used for the comparison between treatments and negative control. (****) *P* < 0.0001

The toxic effect of compound 1 was evaluated through the hemolytic assay in human erythrocytes (Fig. 4). The compound 1 showed no hemolytic effect at the concentrations of 13.75, 27.5 and 55 µM and showed low hemolysis percentage at 110 µM, standing below 0.5%. Compared with the negative control, it did not show any statistical significance in all the concentrations tested (*p* > 0.0001) which indicates that the compound is not toxic to human erythrocytes.

### Compound 1 inhibit cell proliferation reducing colony formation

**Fig. 5:**
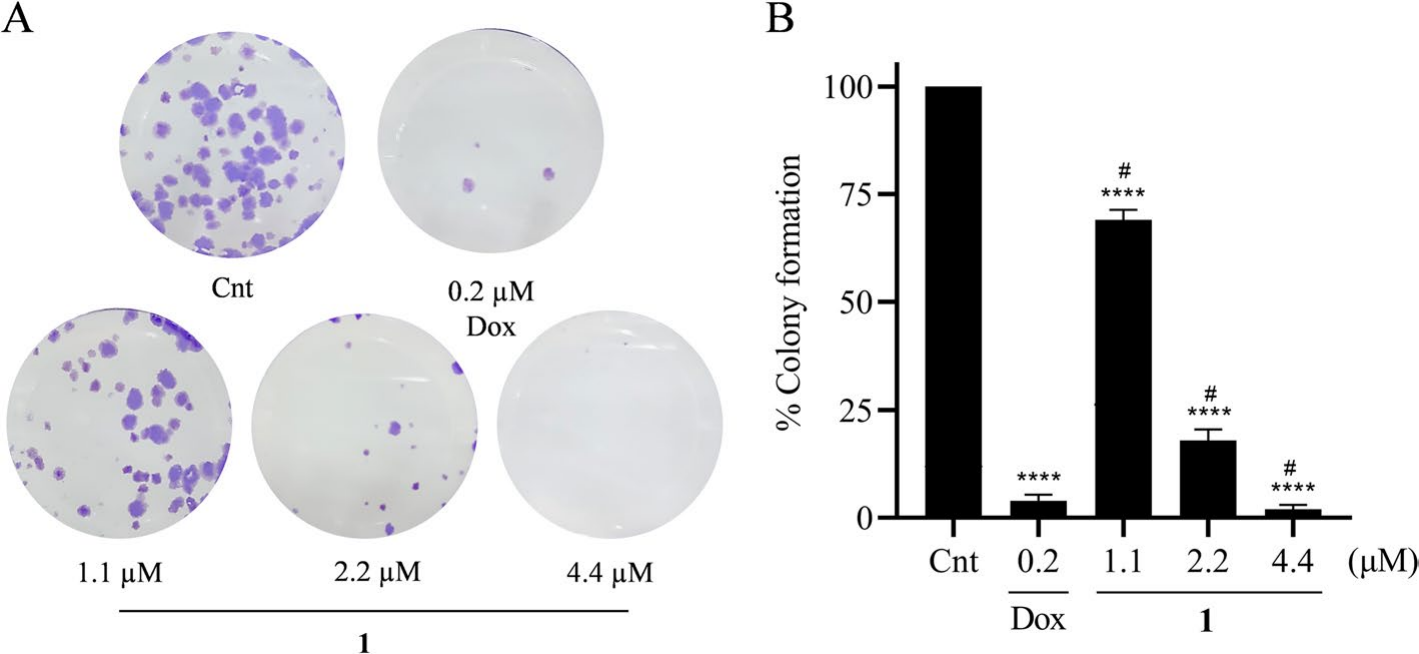
Reduction of A549 colony formation by the compound 1. **A** – Representation of an experiment showing the formation of colonies. **B** – Percentage of colony formation. Cnt = negative control and Dox = doxorubicin. Data were submitted to One-way ANOVA followed by Dunnett's post-test for the comparison between treatments and control and comparison between treatments Tukey’s post-test was used. (****) *P* < 0.001 treatments compared to the negative control and (#) *P* < 0.001 for comparisons between treatments

The ability of compound 1 to inhibit colony formation was evaluated through clonogenic assay. In this assay, A549 cells were treated with the compound 1 at concentrations of 1.1, 2.2 and 4.4 µM for 72 h. After treatment, the compound 1 was able to inhibit the formation of colonies in a concentration-dependent manner. Compound 1 at a concentration of 1.1 µM was able to inhibit colony formation by 31.1%, at a concentration of 2.2 µM the compound was able to inhibit 82.1% while at a concentration of 4.4 µM the compound was able to inhibit 98.1% compared to the negative control (p < 0.001). Doxorubicin inhibited 96.1% of colony formation, similar to the higher concentration of the compound 1 (Fig. 5).

### Compound 1 reduces wound closure after treatment

**Fig. 6:**
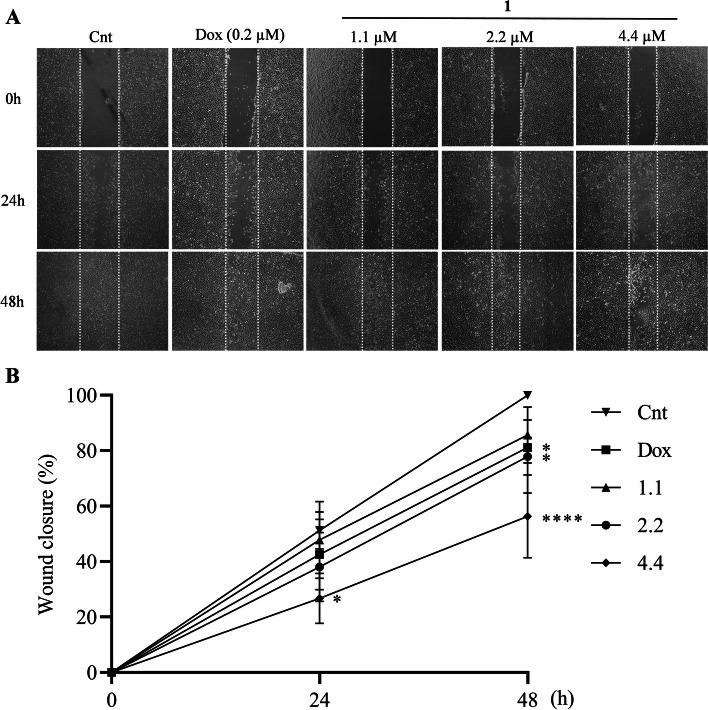
Inhibitory effect on migration of A549 cells after treatment with compound 1. **A** – Representative experiment of a wound-healing after treatment with compound 1 for 0 h, 24 h and 48 h. **B** – Percentage of wound closure. Data shows mean and SD of three independent treatments. Cnt = negative control and Dox = doxorubicin. Data were submitted to Two-way ANOVA followed by Dunnett's post-test was used for the comparison between treatments. (****) *P* < 0.001 and (*) *P* < 0.01

The assay was performed to assess the ability of the compound to inhibit cell migration. After performing the wound, it was possible to observe that the compound 1 significantly inhibited cell migration in A549 cells. In the concentration of 1.1 µM no significant reduction of migration was observed in 24 or 48 h. However, at 2.2 µM, the compound was able to inhibit cell migration, reducing the wound closure by 33% at 48 h. The concentration of 4.4 µM showed the highest inhibitory capacity, with only 18% wound closure at 24 h and 56% at 48 h. In the negative control, at the end of the experiment, the wound was closed in 99% while the positive, doxorubicin, 78% of the wound was restored in 48 h (Fig. 6).

### Compound 1 induces apoptosis and necrosis in A549 cells after treatment

**Fig. 7:**
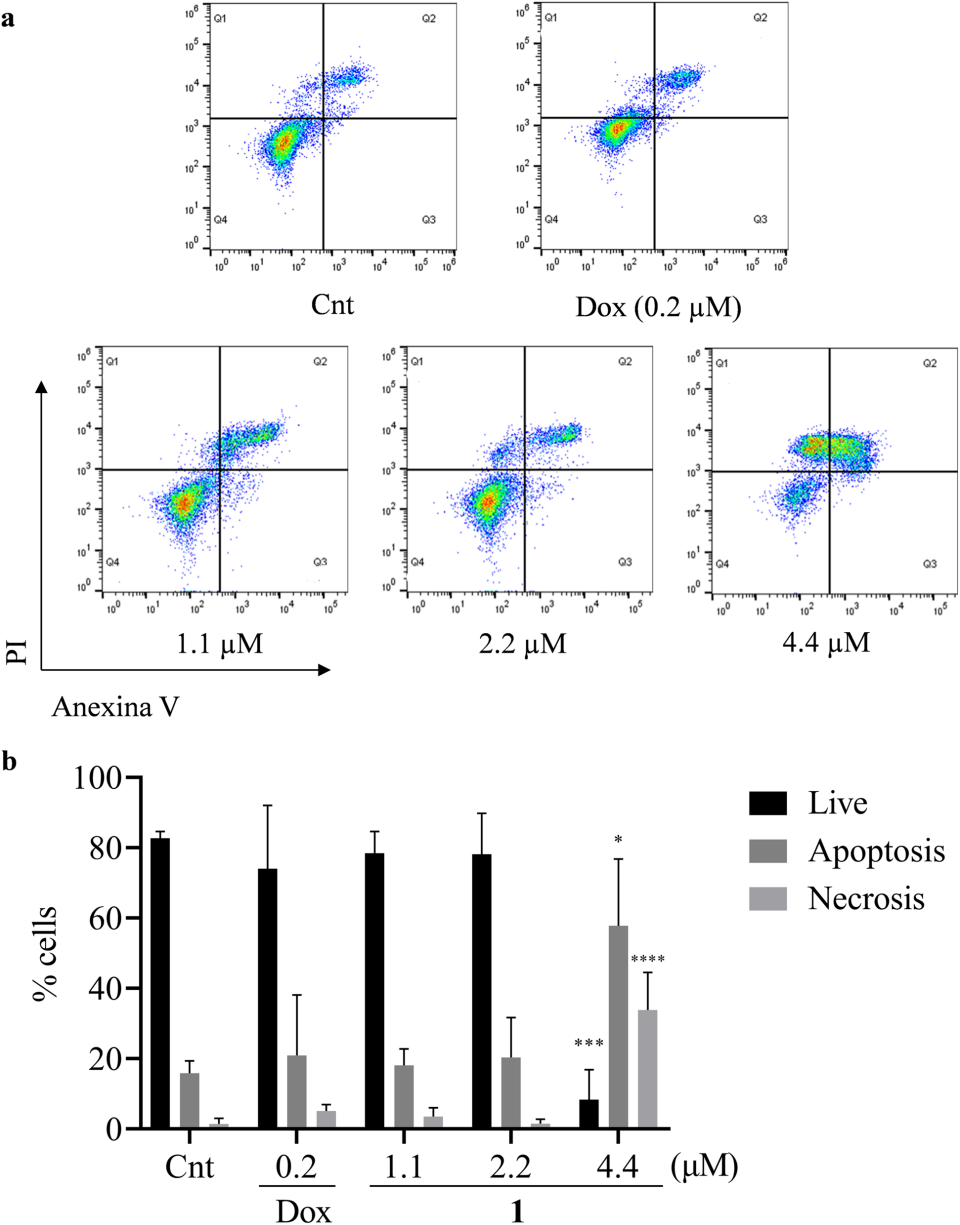
Percentage of A549 cells undergoing apoptosis and necrosis after treatment with compound 1. **A** – Representation of the analysis by flow cytometry. In the quadrants: viable cells (Annexin V − /PI-), early apoptosis (Annexin V + /PI-), late apoptosis (Annexin V + /PI +) and necrosis (Annexin V − /PI +). **B** – Percentage of viable cells in apoptosis and necrosis after treatment with compound 1. Cnt = negative control and Dox = doxorubicin. Data were subjected to statistical analysis One-way ANOVA followed by Dunnett's post-test to compare treatments with the negative control. (****) *P* > 0.0001, (***) *P* < 0.001, and (*) < 0.01

To analyze the possible type of death induced by the compound 1, the Annexin V FITC/PI assay was performed. After treatment for 24 h, it was possible to observe at the concentration of 4.4 µM a significant reduction in the percentage of living cells (12.4%), as well as an increase in cells undergoing apoptosis (47.8%) and necrosis (39.7%) compared to the negative control. The 1.1 µM and 2.2 µM concentrations were not able to induce significant changes in the percentage of living, apoptotic or necrotic cells. It was also not possible to observe changes in the 0.2 µM concentration of doxorubicin (Fig. 7).

## Discussion

Lawsone has been a source of inspiration for the production of derivatives in order to improve its cytotoxic potential [21]. In this work, lawsone derivatives were evaluated for their cytotoxic activity in lung adenocarcinoma (A549), melanoma (B16-F10) and glioma (C6) cell lines. SRB results show that the three compounds were able to inhibit cell growth in the tested cell lines. Among them, compound 1 showed the greatest inhibitory potential compared to compounds 2 and 3, showing IC_50_ values below 2.2 µM in the three cell lines. It is important to notice that compound 2 and 3 showed a high variability of data, which could potentially be attributed to the solubility or structural degradation of these compounds. Consequently, it is possible that these compounds may be excluded from future assessments. In addition, compound 1 stands out for having an inhibitory potential at least 10 times greater than lawsone. Compounds considered potent in cancer treatment must have an IC_50_ below 10 µM after 72 h of treatment [28]. The ability of a compound to be cytotoxic in types of cancer with distinct histological characteristics makes it promising in the treatment of neoplasms [29]. The compound 1 was able to inhibit the cell growth of three histologically different types of cancer, it is suggested that it has great cytotoxic potential. Similar results with glycosidic derivatives of lawsone show effect when used against glioma, carcinoma and melanoma and different breast cancer cell lines [22, 24].

Based on our results and the data found in the literature, some synthetic derivatives show a higher inhibitory effect than their original compound. Although compound 1 showed a high inhibitory effect in all the cancer cell lines tested, the results in the hemolysis assay demonstrated that this compound was not toxic to erythrocytes even in the highest concentration tested of 110 µM. Erythrocytes are constituted by a fragile cell membrane that may be easily damaged by drug activity and it is regularly used as an indicator of toxicity against non-tumoral cells [25, 30, 31]. It reveals that compound 1 has a high inhibitory effect in tumoral cells while shows low cytotoxicity in non-tumoral cells. Therefore, compound 1 was selected for evaluation in the other cytotoxicity assays.

Recently the importance of a terminal alkyne moiety for the activity of some compounds has been the subject of attention of several groups. It has been suggested that the alkyne can serve as a latent electrophile. We speculate that the propargyl group in compound 1 (*O*-propargyllawsone) can contribute to the activity of this compound by in vitro alkylation of important biomolecules such as the cysteine protease cathepsin K (CatK) by forming an irreversible covalent bond between the active-site cysteine thiol and the internal carbon of the alkyne moiety [32]. CatK is known to be upregulated in many types of cancer which promotes cell proliferation, invasion and migration and its inhibition has been demonstrated as an approach to cancer treatment [33, 34].

Tumor cells have high proliferation rates due to the accumulation of mutations that favor cell division and reduce death. A single mutated cell can divide countless times, forming tumors, which is one of the important aspects for the establishment of cancer [35]. The clonogenic assay allows evaluating the capacity of a cell to resist the exposed treatment and to proliferate forming colonies [36]. In this study, it was possible to observe that the compound 1 significantly inhibited the formation of colonies in the lung carcinoma at all concentrations tested. The inhibitory effect of tumor clones by compound 1 corroborates the results of cytotoxicity by SRB in which the compound showed a high inhibitory potential.

Studies with the compound 1,4-naphthoquinone demonstrated that this naphthoquinone was able to inhibit the formation of melanoma colonies [37]. Sadhukhan [38] tested a total of twenty-two lawsone-derived compounds in human glioblastoma multiforme lines. They observed that one of the compounds was able to inhibit colony formation compared to the negative control. β-lapachone, a lawsone-like naphthoquinone, can inhibit the formation of colonies in the lung carcinoma by its ability to produce reactive oxygen species [39]. Thus, compounds derived from lawsone have a high capacity to inhibit the formation of tumor cell colonies.

The 1,4-naphthoquinone antitumor activity is generally attributed to a variety of mechanisms which may differ depending on the cell type. Among Others, the redox potential of these compounds, leads to the production of reactive oxygen species (ROS) via one-electron-reductase-mediated redox cycling [40]. This can account, at least in part, for the activity of the compounds described herein.

Lung adenocarcinoma is a cancer with a high mortality rate and highly metastatic, in which cell migration plays an important role in tumor progression [41, 42]. Compounds with the ability to prevent proliferation are of great relevance for the treatment of this cancer [43]. As observed in the wound-healing assay, the compound 1 has a high capacity to inhibit cell migration in lung carcinoma. These results corroborate the data shown above, making the compound antiproliferative capacity evident. Literature results show that lawsone derivatives, like those studied in this work, also demonstrate the ability to inhibit cell migration. Hong [44] demonstrated that a naphthoquinone known as shikonin also showed a high inhibitory effect in cell migration in breast cancer. Oliveira [21] observed that ruthenic compounds complexed with lawsone were also able to inhibit cell migration. After 48 h these compounds inhibited about 70% of cell migration in the lung adenocarcinoma lineage.

The morphological changes observed in apoptosis may be the loss of the fibroblast aspect or cell rounding, reduction of the cytoplasmic volume, usually accompanied by the condensation and fragmentation of the nucleus [45]. In the results obtained by Giemsa staining, the compound 1 induced morphological changes characteristic of apoptosis in A549, such as cytoplasm reduction and cells with a round appearance, indicating that the compound may induce apoptosis.

Activation of caspase pathways in apoptosis induces exposure to membrane phospholipids such as phosphatidylserine [46]. Phosphatidylserine is found inside the lipid bilayer and after induction of apoptosis, it is externalized in the plasma membrane where it will serve as a phagocytosis signal for cells such as macrophages [47, 48]. The results found in the Annexin V/PI assay showed that at a concentration of 4.4 µM, the compound 1 may induce cell death by apoptosis and necrosis. This result corroborates the morphological analysis observed by Giemsa staining, in which alterations such as reduced cytoplasm and apoptotic cells were seen.

Lawsone and several other quinones induce death by apoptosis in a variety of tumor cells. One of the apoptosis mechanisms described for naphthoquinones is the inactivation of topoisomerases I and II, as demonstrated in the in vitro and in vivo glioma model (C6), in which lapachol was able to induce apoptosis after treatment [49]. Lawsone-containing ruthenium complexes induce apoptosis in prostate cancer cells, breast cancer and lung cancer with cell cycle arrest in the G1 phase [21]. Glycosidic derivative of lawsone was able to induce apoptosis in melanoma cells, however the mechanism of action has not yet been described [24].

## Conclusions

The results obtained suggest that the compound *O*-propargyllawsone was able to inhibit cell growth, as well as induce morphological changes in the A549 cells. The compound also inhibited the formation of clones in the A549 and the death mechanism is suggested to be apoptosis. Although further evaluation of the mechanism of action is needed, the *O*-propargyllawsone seems to be a promising compound in the treatment of lung adenocarcinoma.

### Supplementary Information

**Additional file 1:** **Fig S1.** Percentage of growth inhibition of Doxorubicin. Results obtained after 72h treatment in A549 and B16-F10 cell lines. **Fig S2.** Percentage of growth inhibition of Lawsone. Results obtained after 72h treatment in A549 and C6 cell lines.

## Data Availability

All data generated or analyzed during this study are included in this published article.

## Abbreviations

CatK: Cysteine Protease Cathepsin K
DMEM: Dulbeccos's Modified Eagle Medium
DMSO: Dimethyl Sulfoxide
EDTA: Ethylenediaminetetraacetic Acid
FBS: Fetal Bovine Serum
GI: Growth Inhibition
IC: Inhibitory Concentration
NFκB: Nuclear Factor-κB
p53: Tumor Protein p53 Gene
PBS: Phosphate-Buffered Saline
PI: Propidium Iodide
ROS: Reactive Oxygen Species
SRB: Sulforhodamine B

## References

[1] Sung H, Ferlay J, Siegel RL, Laversanne M, Soerjomataram I, Jemal A (2021). Global Cancer Statistics 2020: GLOBOCAN Estimates of Incidence and Mortality Worldwide for 36 Cancers in 185 Countries. CA Cancer J Clin.

[2] Herbst RS, Morgensztern D, Boshoff C (2018). The biology and management of non-small cell lung cancer. Nat.

[3] Ding L, Getz G, Wheeler DA, Mardis ER, McLellan MD, Cibulskis K (2008). Somatic mutations affect key pathways in lung adenocarcinoma. Nat.

[4] Friedlaender A, Banna G, Malapelle U, Pisapia P, Addeo A (2019). Next generation sequencing and genetic alterations in squamous cell lung carcinoma: Where are we today?. Front Oncol.

[5] Heidemann F, Schildt A, Schmid K, Bruns OT, Riecken K, Jung C (2014). Selectins mediate small cell lung cancer systemic metastasis. PLoS ONE.

[6] Chang A (2011). Chemotherapy, chemoresistance and the changing treatment landscape for NSCLC. Lung Cancer.

[7] Efferth T, Saeed MEM, Kadioglu O, Seo EJ, Shirooie S, Mbaveng AT, et al. Collateral sensitivity of natural products in drug-resistant cancer cells. Biotechnol Adv. 2019. 10.1016/j.biotechadv.2019.01.009. 10.1016/j.biotechadv.2019.01.009 30708024

[8] Fitsiou E, Mitropoulou G, Spyridopoulou K, Tiptiri-Kourpeti A, Vamvakias M, Bardouki H (2016). Phytochemical profile and evaluation of the biological activities of essential oils derived from the greek aromatic plant species Ocimum basilicum, Mentha spicata Pimpinella anisum and Fortunella margarita. Molecules.

[9] Fitsiou E, Mitropoulou G, Spyridopoulou K, Vamvakias M, Bardouki H, Galanis A, et al. Chemical composition and evaluation of the biological properties of the essential oil of the dietary phytochemical Lippia citriodora. Mol. 2018;23:123. 10.3390/molecules23010123. 10.3390/molecules23010123 PMC6017519 29329229

[10] Guesmi F, Prasad S, Tyagi AK, Landoulsi A (2017). Antinflammatory and anticancer effects of terpenes from oily fractions of Teucruim alopecurus, blocker of IκBα kinase, through downregulation of NF-κB activation, potentiation of apoptosis and suppression of NF-κB-regulated gene expression. Biomed Pharmacother.

[11] Newman DJ, Cragg GM (2020). Natural Products as Sources of New Drugs over the Nearly Four Decades from 01/1981 to 09/2019. J Nat Prod.

[12] Demain AL, Vaishnav P (2011). Natural products for cancer chemotherapy. Microb Biotechnol.

[13] Verma R (2006). Anti-Cancer Activities of 1,4-Naphthoquinones: A QSAR Study. Anticancer Agents Med Chem.

[14] Li P, Xin H, Liu W (2017). Lawsone inhibits cell growth and improves the efficacy of cisplatin in skov-3 ovarian cancer cell lines. Afr J Tradit Complement Altern Med.

[15] Rahmoun NM, Boucherit-Otmani Z, Boucherit K, Benabdallah M, Villemin D, Choukchou-Braham N (2012). Antibacterial and antifungal activity of lawsone and novel naphthoquinone derivatives. Med Mal Infect.

[16] Dananjaya SHS, Udayangani RMC, Shin SY, Edussuriya M, Nikapitiya C, Lee J (2017). In vitro and in vivo antifungal efficacy of plant based lawsone against Fusarium oxysporum species complex. Microbiol Res.

[17] Wang S bin, Tao Z, Li P. Lawsone suppresses azoxymethane mediated colon cancer in rats and reduces proliferation of DLD-1 cells via NF-κB pathway. Biomed Pharmacother. 2017;89: 152–161. 10.1016/j.biopha.2017.01.169. 10.1016/j.biopha.2017.01.169 28222396

[18] Li P, Xin H, Liu W (2017). Lawsone inhibits cell growth and improves the efficacy of cisplatin in skov-3 ovarian cancer cell lines. Afr J Tradit Complement Altern Med.

[19] Barani M, Mirzaei M, Torkzadeh-Mahani M, Nematollahi MH (2018). Lawsone-loaded Niosome and its antitumor activity in MCF-7 breast Cancer cell line: a Nano-herbal treatment for Cancer. DARU, J Pharm Scie.

[20] de Grandis RA, Santos PW da S dos, Oliveira KM de, Machado ART, Aissa AF, Batista AA, et al. Novel lawsone-containing ruthenium(II) complexes: Synthesis, characterization and anticancer activity on 2D and 3D spheroid models of prostate cancer cells. Bioorg Chem. 2019;85: 455–468. 10.1016/j.bioorg.2019.02.010. 10.1016/j.bioorg.2019.02.010 30776556

[21] Oliveira KM, Liany LD, Corrêa RS, Deflon VM, Cominetti MR, Batista AA (2017). Selective Ru(II)/lawsone complexes inhibiting tumor cell growth by apoptosis. J Inorg Biochem.

[22] Ottoni FM, Gomes ER, Pádua RM, Oliveira MC, Silva IT, Alves RJ (2020). Synthesis and cytotoxicity evaluation of glycosidic derivatives of lawsone against breast cancer cell lines. Bioorg Med Chem Lett..

[23] Hamdoun S, Fleischer E, Klinger A, Efferth T (2017). Lawsone derivatives target the Wnt/β-catenin signaling pathway in multidrug-resistant acute lymphoblastic leukemia cells. Biochem Pharmacol.

[24] de Franca MNF, Isidório RG, Bonifacio JHO, dos Santos EWP, Santos JF, Ottoni FM (2021). Anti-proliferative and pro-apoptotic activity of glycosidic derivatives of lawsone in melanoma cancer cell. BMC Cancer.

[25] Andrade LN, Lima TC, Amaral RG, do Ó Pessoa C, de Moraes Filho MO, Soares BM, et al. Evaluation of the cytotoxicity of structurally correlated p-menthane derivatives. Mol. 2015;20: 13264–13280. 10.3390/molecules200713264. 10.3390/molecules200713264 PMC6331850 26197313

[26] Samarghandian S, Shabestari MM (2013). DNA fragmentation and apoptosis induced by safranal in human prostate cancer cell line. Indian J Urol.

[27] Mahmoud TS, Marques MR, do Ó Pessoa C, Lotufo LVC, Magalhães HIF, de Moraes MO, et al. In vitro cytotoxic activity of brazilian middle west plant extracts. Brazilian J Pharm. 2011;21: 456–464. 10.1590/S0102-695X2011005000061.

[28] Kuete V, Ango PY, Yeboah SO, Mbaveng AT, Mapitse R, Kapche GDWF, et al. Cytotoxicity of four Aframomum species (A. arundinaceum, A. alboviolaceum, A. kayserianum and A. polyanthum) towards multi-factorial drug resistant cancer cell lines. BMC Complement Altern Med. 2014;14: 1–7. 10.1186/1472-6882-14-340. 10.1186/1472-6882-14-340 PMC4177760 25239700

[29] Carvalho C, Santos R, Cardoso S, Correia S, Oliveira P, Santos M (2009). Doxorubicin: The Good, the Bad and the Ugly Effect. Curr Med Chem.

[30] Sharma P, Sharma JD. In vitro hemolysis of human erythrocytes-by plant extracts with antiplasmodial activity. J Ethnopharmacol. 2001. Available: www.elsevier.com/locate/jethpharm. 10.1016/s0378-8741(00)00370-6 11274824

[31] Pita JCLR, Xavier AL, De Sousa TKG, Mangueira VM, Tavares JF, De Oliveira RJ (2012). In Vitro and in Vivo antitumor effect of trachylobane-360, a diterpene from Xylopia langsdorffiana. Molecules.

[32] Mons E, Jansen IDC, Loboda J, van Doodewaerd BR, Hermans J, Verdoes M (2019). The Alkyne Moiety as a Latent Electrophile in Irreversible Covalent Small Molecule Inhibitors of Cathepsin K. J Am Chem Soc.

[33] Qian D, He L, Zhang Q, Li W, Tang D, Wu C, et al. Cathepsin K: A versatile potential biomarker and therapeutic target for various cancers. Curr Oncol MDPI. 2022: 5963–5987. 10.3390/curroncol29080471. 10.3390/curroncol29080471 PMC9406569 36005209

[34] Liang W, Wang F, Chen Q, Dai J, Escara-Wilke J, Keller ET (2019). Targeting cathepsin K diminishes prostate cancer establishment and growth in murine bone. J Cancer Res Clin Oncol.

[35] Frank SA, Nowak MA (2004). Problems of somatic mutation and cancer. BioEssays.

[36] Guzmán C, Bagga M, Kaur A, Westermarck J, Abankwa D (2014). ColonyArea: An ImageJ plugin to automatically quantify colony formation in clonogenic assays. PLoS ONE.

[37] Kumar MRS, Aithal K, Rao BN, Udupa N, Rao BSS (2009). Cytotoxic, genotoxic and oxidative stress induced by 1,4-naphthoquinone in B16F1 melanoma tumor cells. Toxicol In Vitro.

[38] Sadhukhan P, Saha S, Sinha K, Brahmachari G, Sil PC (2016). Selective pro-apoptotic activity of novel 3,3′-(aryl/alkyl-methylene)bis(2-hydroxynaphthalene-1,4-dione) derivatives on human cancer cells via the induction reactive oxygen species. PLoS ONE.

[39] Choi EK, Terai K, Ji IM, Kook YH, Park KH, Oh ET (2007). Upregulation of NAD(P)H:quinone oxidoreductase by radiation potentiates the effect of bioreductive β-lapachone on cancer cells. Neoplasia.

[40] Pereyra CE, Dantas RF, Ferreira SB, Gomes LP, Silva FP. The diverse mechanisms and anticancer potential of naphthoquinones. Cancer Cell International. BioMed Central Ltd. 2019. 10.1186/s12935-019-0925-8. 10.1186/s12935-019-0925-8 PMC6679553 31388334

[41] Hung JY, Horn D, Woodruff K, Prihoda T, Lesaux C, Peters J (2014). Colony-stimulating factor 1 potentiates lung cancer bone metastasis. Lab Invest.

[42] Zaballa I, Eidemüller M (2016). Mechanistic study on lung cancer mortality after radon exposure in the Wismut cohort supports important role of clonal expansion in lung carcinogenesis. Radiat Environ Biophys.

[43] Hittelman WN (1999). Clones and subclones in the lung cancer field. J Natl Cancer Inst.

[44] Hong D, Jang SY, Jang EH, Jung B, Cho IH, Park MJ (2015). Shikonin as an inhibitor of the LPS-induced epithelial-to-mesenchymal transition in human breast cancer cells. Int J Mol Med.

[45] Doonan F, Cotter TG (2008). Morphological assessment of apoptosis. Methods.

[46] Fiandalo MV, Kyprianou N (2012). Caspase control: protagonists of cancer cell apoptosis. Exp Oncol.

[47] Mariño G, Kroemer G (2013). Mechanisms of apoptotic phosphatidylserine exposure. Cell Res.

[48] van den Eijnde SM, Boshart L, Baehrecke EH, de Zeeuw CI, Reutelingsperger CPM, Vermeij-Keers C (1998). Cell surface exposure of phosphatidylserine during apoptosis is phylogenetically conserved. Apoptosis.

[49] Xu H, Chen Q, Wang H, Xu P, Yuan R, Li X (2016). Inhibitory effects of lapachol on rat C6 glioma in vitro and in vivo by targeting DNA topoisomerase i and topoisomerase II. J Exp Clin Cancer Res.

