# Supplementary material for: Inhibitory effect of O-propargyllawsone in A549 lung adenocarcinoma cells
Source: BMC Complement Med Ther. 2023 Sep 20;23:333. doi: 10.1186/s12906-023-04156-9 (PMC10510246; doi:10.1186/s12906-023-04156-9)
Supplement: Supplementary file 1 — Additional file 1: Fig S1. Percentage of growth inhibition of Doxorubicin. Results obtained after 72h treatment in A549 and B16-F10 cell lines. Fig S2. Percentage of growth inhibition of Lawsone. Results obtained after 72h treatment in A549 and C6 cell lines. [file 12906_2023_4156_MOESM1_ESM.docx]

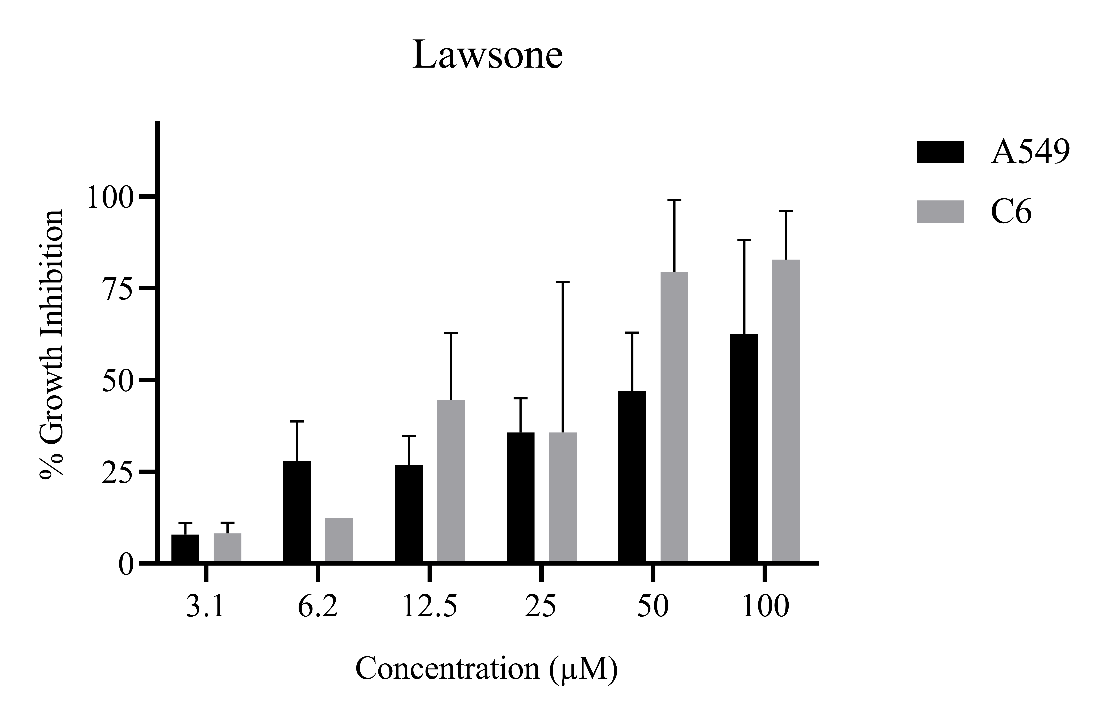


**Fig S2. Percentage of growth inhibition of Lawsone.** Results obtained after 72h treatment in A549 and C6 cell lines.


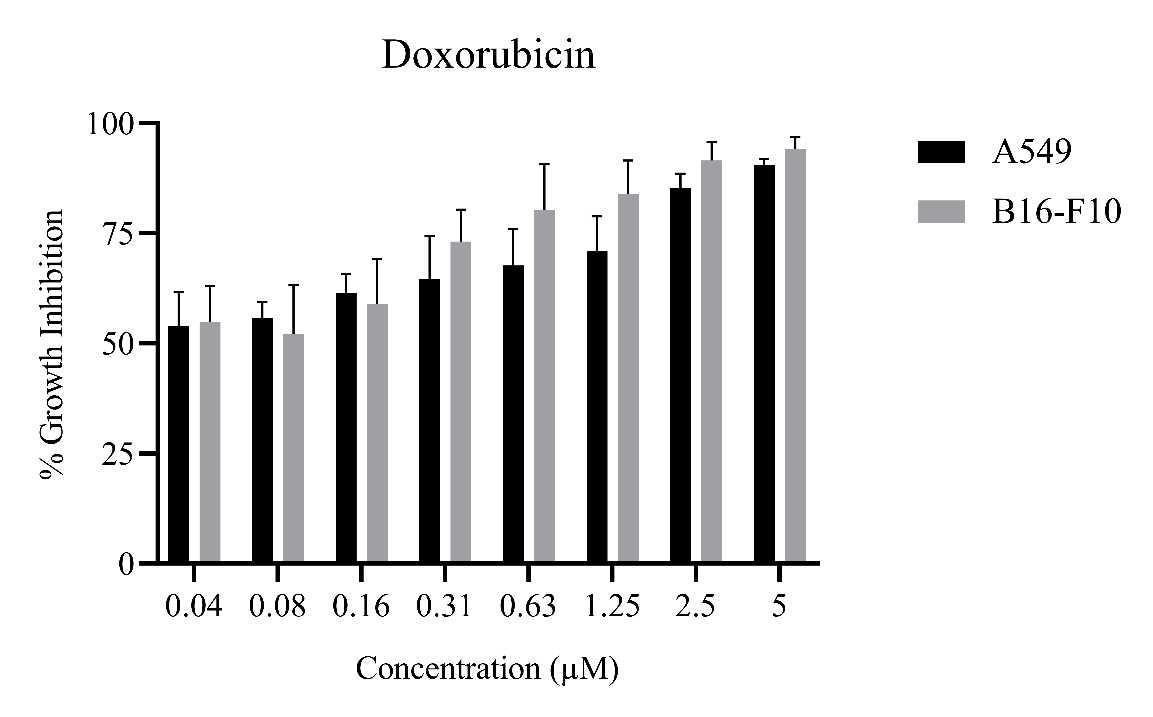


**Fig S1. Percentage of growth inhibition of Doxorubicin.** Results obtained after 72h treatment in A549 and B16-F10 cell lines.
